# The Study of Clinical and Biochemical Parameters in Assessing the Response to the Antiviral Therapy in the Chronic Viral Hepatitis B

**DOI:** 10.3390/medicina57080757

**Published:** 2021-07-26

**Authors:** Alice Elena Ghenea, Vlad Pădureanu, Ramona Cioboată, Anca-Loredana Udriștoiu, Andrei Ioan Drocaş, Eugen Țieranu, Mara Carsote, Corina Maria Vasile, Aritina Moroşanu, Viorel Biciușcă, Alex-Ioan Salan, Adriana Turculeanu, Anca Ungureanu

**Affiliations:** 1 Department of Bacteriology-Virology-Parasitology, University of Medicine and Pharmacy of Craiova, 200349 Craiova, Romania; gaman_alice@yahoo.com (A.E.G.); adriana_turculeanu@yahoo.com (A.T.); ancaungureanu65@yahoo.com (A.U.); 2 Department of Internal Medicine, University of Medicine and Pharmacy of Craiova, 200349 Craiova, Romania; biciuscaviorel@gmail.com; 3 Department of Pneumology, University of Pharmacy and Medicine Craiova, 200349 Craiova, Romania; ramona.cioboata@umfcv.ro; 4 Faculty of Automation, Computers and Electronics, University of Craiova, 200776 Craiova, Romania; anca.udristoiu@edu.ucv.ro; 5 Department of Urology, University of Medicine and Pharmacy of Craiova, 200349 Craiova, Romania; andrei_drocas@yahoo.com; 6 Department of Cardiology, University of Medicine and Pharmacy of Craiova, 200349 Craiova, Romania; tieranueugen@gmail.com; 7 Department of Endocrinology, C.I. Parhon National Institute of Endocrinology, Carol Davila University of Medicine and Pharmacy, 050474 Bucharest, Romania; carsote_m@hotmail.com; 8 Department of Pediatric Cardiology, County Clinical Emergency Hospital of Craiova, 200349 Craiova, Romania; corina.vasile93@gmail.com; 9 Department of Paediatrics, University of Medicine and Pharmacy Craiova, 200349 Craiova, Romania; inamorosanu@yahoo.com; 10 Department of Oral and Maxillofacial Surgery, University of Medicine and Pharmacy Craiova, 200349 Craiova, Romania; alex.salan@umfcv.ro

**Keywords:** antiviral therapy, chronic hepatitis B, interferon-alpha α-2a/b

## Abstract

*Background and Objectives*: Hepatitis B virus infection remains a major public health concern. The interaction between hepatitis B virus (HBV) hepatitis B virus and the host inflammatory response is an important contributing factor driving liver damage and diseases outcomes. The management of chronic hepatitis B virus infection is an area of massive unmet clinical need worldwide. Our primary aim for this study was to evaluate biological response rates and sustained virological response in patients with chronic hepatitis B treated with Peg-IFN α-2a/b. The second aim of the study was the identification of metabolic changes and insulin resistance. *Materials and Methods*: We enrolled in this study 166 patients who fulfilled all inclusion and exclusion criteria. These treatment-naive patients with chronic HBV were treated with Pegylated Interferon α-2a/b. HBV infection was defined by the presence of HBV serological markers (HBsAg, anti-HBsAb, anti-HBcAb, HBeAg, anti HBeAb) by Enzyme-Linked Immuno Sorbent Assay (ELISA) and serum HBV-DNA levels were estimated by a commercially available quantitative polymerase chain reaction (PCR) assay. *Results*: Patients’ recovery progress has been evaluated by determining the following: age, gender; biochemical tests; alanine aminotransferase, aspartate aminotransferase; serological assays for HBV serological markers (HBsAg, anti-HBsAc/Ab, anti-HBcAc/Ab, HBeAg, anti HBeAc/Ab); molecular tests to detect viral particles, testing for HBV DNA (PCR) to confirm the diagnosis and quantify the number of viral copies in the blood (viremia); liver ultrasound—performed through epigastric and intercostal approach (transversal and longitudinal sections). *Conclusions*: Our results indicated that only HOMA index values, that of fasting insulin, together with baseline HBV DNA, alanine aminotransferase values, mean blood glucose at the beginning of treatment may be predictive of the early viral response in chronic hepatitis B.

## 1. Introduction

Hepatitis B infection progresses to acute/chronic hepatitis, severe liver failure and death, and it is still a significant global health concern, although there are treatment strategies and effective vaccines. Two billion people have been infected with HB worldwide. It is estimated that more than 292 million people are living with chronic hepatitis B (CHB) infection worldwide [1]. Annually, 887,000 deaths occur each year due to HB and related illnesses [2]. Although chronic infection develops in approximately 90% of infants, 30–50% of children aged five years, and 5–10% adults [3,4,5,6]. Especially in low- and middle-income countries, hepatitis B virus (HBV) infection still remains a serious global public health. Acute hepatitis B infection progresses in a proportion of patients into chronicity. Chronic hepatitis B virus (HBV) infection is a globally challenging disease; it subsequently increases the risk of liver cirrhosis and hepatocellular carcinoma (HCC). Even without the presence of cirrhosis, patients with CHB could occur HCC [1]. Annually, more than one million patients with CHB die due to liver failure or HCC [2]. Non-alcoholic fatty liver disease (NAFLD) represents an ensemble of liver damage that includes simple steatosis, non-alcoholic steato-hepatitis (NASH), fibrosis, and cirrhosis. It is a type of liver injury caused by metabolic stress that is intimately tied to insulin resistance and hereditary predisposition. NAFLD not only can directly lead to hepatic impairment and liver-related death, but can increase the risk of obesity, metabolic syndrome (MetS), cardiovascular disease, and type 2 diabetes mellitus. In recent years, NAFLD has quickly become the most common chronic liver disease globally, along with lifestyle changes and standard of living improvements. This is accompanied by NASH (specifically) in the USA, where people awaiting liver transplantation constitute the majority of patients [7]. Metabolic syndrome (MetS) has various definitions; however, all these definitions stress the presence of abdominal obesity in conjunction with other parameters [8,9]. Insulin resistance can be defined as a subnormal biological response to normal insulin concentrations. The underlying causes are generally obesity, stress-related contraregulatory hormone imbalance, lipodystrophy, and various drugs. It is frequently accompanied by diabetes, metabolic syndrome, and nonalcoholic fatty liver disease (NAFLD), in which insulin resistance is the key mechanism and found to be positive above 75%. Insulin resistance can be practically measured with the HOMA-IR score. For development of metabolic syndrome and type 2 diabetes, insulin resistance is a principal indication. In addition, insulin resistance appears as a consequence of the inability of insulin to induce the appropriate effect on glucose metabolism. Inordinately large amounts of insulin are required to achieve a normal response in a state of insulin resistance. A hyperinsulinemic state causes clinical abnormalities to appear in the blood vessels, kidneys, and liver, and these represent the characteristics of metabolic syndrome.

The relationship between chronic hepatitis B infection and insulin resistance has been clearly demonstrated and insulin resistance has been identified as a risk factor in liver fibrosis, reduction of the permanent response in interferon alpha-based treatment, and HCC development [10,11]. HBV vaccination is the safest and main precaution from being exposed to the virus [2].

The treatment’s primary aim is to save lives by decreasing liver transplant, liver cancer death, reverse or slow liver disease progression and infectivity [4]. IFN-α have antiviral functions against a variety of viruses and are a host defense against HBV infections by interferon-stimulated genes (ISGs) [11]. In 76–94% of individuals, the treatment response is associated with more confirmatory clinical outcomes in terms of liver-related complications and survival [12]. The first certified treatment choice for CHB infection was IFN-α-2a/b, which was replaced by the standard IFN-α-2b because of pharmacokinetic properties. Pegylation is used in order to increase the half-life of interferon [13]. The objectives of our research were:

- Evaluation of the biological response rates and sustained virological response in patients with chronic hepatitis B treated with PegIFN α-2a/b.
- Identify metabolic changes and insulin resistance.

## 2. Materials and Methods

The research was performed in the Internal Medicine Clinic of Filantropia University Hospital, in 2018–2020. The study group consisted of 166 patients who fulfilled all inclusion and exclusion criteria. These treatment-naive patients with chronic HBV were treated with Pegylated Interferon α-2a/b. HBV infection was defined by the presence of HBV serological markers (HBsAg, anti-HBsAb, anti-HBcAb, HBeAg, anti HBeAb) by Enzyme-Linked Immuno Sorbent Assay (ELISA)- Elecsys HBsAg II (Roche Diagnostics, Indianapolis, IN, USA) and serum HBV-DNA levels were estimated by a commercially available quantitative polymerase chain reaction (PCR) assay-Alinity™ m HBV assay (Abbott Laboratories. Abbott Park, IL, USA) with a dynamic range of quantification from 10 to 109 UI/mL (1.0–9.0 Log IU/mL).

### 2.1. Treatment Protocol

The patients were treated with Pegylated Interferon α-2a 180 mg/week and the duration of the therapy was 48 weeks.

All patients were subjected to:

1. Clinical evaluation; including demographic data, present history of smoking, alcohol consumption, presence of chronic diseases, and past history of dental intervention, surgery or blood transfusion.
  - Coinfection with HIV, HCV or HDV;
  - Previous antiviral treatment for any length of time;
  - Other chronic liver diseases (chronic alcoholism, Wilson disease, NASH, NAFLD) liver cirrhosis, history of ascites, variceal bleeding, hepatic encephalopathy, and other conditions suggesting decompensated liver disease, fasting plasma glucose > 120 mg/dL; neoplasia diagnosed or treated in the last 5 years, patients treated with corticosteroids or immunosuppressants in the last 30 days, or if they are expected to also administer medications during the study, known allergies to antiviral medications
2. Anthropometric data: weight, height, body mass index (BMI), calculated by the formula BMI = W/H2, where W (weight) was expressed in kilograms and H (height) in meters. Subjects according to BMI classification were as follows: normal weight-BMI < 25 kg/m^2^, overweight-BMI = 25–29.9 kg/m^2^, obese-BMI ≥ 30 kg/m^2^.
3. Laboratory investigations;
  - Biochemical tests: alanine aminotransferase (ALT), aspartate aminotransferase (AST) by spectrophotometric method, fasting glucose by spectrophotometric method, fasting insulin by hexokinase method, total cholesterol (TC) and triglycerides (TG) by spectrophotometric method. We determined serum iron (Fe/kg) by spectrophotometric method and also serum ferritin by electrochemiluminescence method (ECLIA).
  - Assessment of insulin sensitivity: We determined insulin sensitivity using HOMA-IR (homeostasis model of insulin resistance) using the following formula: fasting plasma glucose (mg/dL) × fasting insulinemia (μU/mL)/405.
  - Diagnostic tests for the detection of HBV infection:
    ○ Serological assays for HBV serological markers (HBsAg, anti-HBsAc/Ab, anti-HBcAc/Ab, HBeAg, anti HBeAc/Ab) by Enzyme-linked immunosorbent Assay (ELISA)
    ○ Molecular tests to detect viral particles. Testing for HBV DNA (PCR) to confirm the diagnosis and quantify the number of viral copies in the blood (viremia).
4. Liver ultrasound, performed through epigastric and intercostal approach (transversal and longitudinal sections), was recorded through the dimensions of the left and right liver lobe, its echogenicity and structure, also diaphragm visibility, posterior attenuation, blood vessels’ appearance, the portal vein system and the spleen.

### 2.2. Patients

The patients’ samples were enrolled in the study, after obtaining the informed consent of each patient. The patients’ cohort was divided into 2 groups:

1. Responders: patients whose PCR results showed early viral response (EVR).
2. Non-responders: patients whose PCR results did not show EVR. The viral load result of respective patients was collected from patients.

Regarding the assessment of treatment response, the initial response was evaluated at 6 months of therapy by determining:

- ALT serum levels
- HBV-DNA. If it has not decreased by more than 2log10, it is considered primary resistance and stops treatment.

Subsequently, we checked periodically, every six months:

- ALT;
- AgHBs;
- AgHBe;
- Anti-Hbe needle;
- HBV-DNA.

Depending on the biochemical and virological response, treatment will stop or continue for up to 5 years. Increased transaminases during treatment requires viremia, and increased viremia under treatment is considered resistance and lack of therapeutic response.

Resistance and lack of response require reassessment of the patient and a new therapeutic decision. The emergence of HBs anti-needle requires stopping therapy.

### 2.3. Statistical Analysis

For storing the information registered on the plug of study in a database and also for statistical calculations, we used statistical software MedCalc^®^ version 20.009 (MedCalc Software, Ostend, Belgium). Statistical test results will be represented by probability hypothesis “null” (p); its value below 0.05 shows a statistically significant difference between the groups studied.

Statistical analysis was performed according to the protocol of a randomized clinical trial on 2 groups of patients having comparable age, sex, etiology of disease, laboratory and histologic parameters.

The parameters studied were included in an AUC (area under curve) analysis to estimate their degree of influence on the response to treatment. Comparison of averages between two groups was made using the Student’s *t*-test, for continuous variables with normal distribution, and the Mann–Whitney U-test for continuous variables with non-normal distribution. 

For comparing the averages between groups, we used the analysis of variance (ANOVA), for continuous variables with normal distribution and for variables with non-normal distribution the Kruskal–Wallis test, respecting the conditions of independence, normality and homeoscedasticity of the lots. The Kruskal–Wallis test was also applied for 95% confidence.

For the analysis of categorical variables, qualitative variables, the Chi-squared-X2 or Fisher’s Exact Test, were applied. 

## 3. Results

The group consisted of 166 patients, as shown in Table 1, with patients with chronic hepatitis B of which 72 women with a mean age of 44.3 years, mean weight 66.9 kg and 94 men with a mean age of 45.3 years, mean weight 70.7 kg. We found a body mass index in women ranged between 22.5 and 25.1, with a mean value of 23 and in males between 23.3 and 25.6, with a mean value of 24.5. 

Among the biochemical analyzed parameters, glycaemia also is included, aiming its fasting value. We followed the fasting glucose values and recorded an average of 99.82 mg/dl in women, 98.0 mg/dL in men. ALT was also measured, resulting in average values of 154 U/L in women and 143.1 U/L in men. Likewise, AST was measured with mean values of 135 U/L in women and 119 U/L in men. Viremia, important on the response to treatment, was analyzed in our group of patients, finding in women a mean viral load of 3,425,458 UI/mL and in men 3,681,361 UI/mL.

US B-mode imaging allows to subjectively estimate the degree of fatty infiltration in the liver. The grading of liver steatosis is usually obtained using some US features that include liver brightness, contrast between the liver and the kidney, US appearance of the intrahepatic vessels, liver parenchyma and diaphragm. Steatosis is graded as follows:

- Absent (score 0) when the echotexture of the liver is normal;
- Mild (score 1), diffusely increased hepatic echogenicity but periportal and diaphragmatic echogenicity is still appreciable;
- Moderate (score 2), diffusely increased hepatic echogenicity obscuring periportal echogenicity but diaphragmatic echogenicity is still appreciable;
- Severe (score 3), in case of marked increase of liver echogenicity with poor or no visualization of portal vein wall, diaphragm, and posterior part of the right liver lobe [14,15,16].

Patients in the study who were unresponsive to treatment (50) had lower average steatosis score (2.62) than others (2.80).

The study of clinical and biochemical factors involved 6 months’ viral response. 

### 3.1. Influence of Cytolysis Enzyme Values

Biochemical response is defined as normalization of ALT level. It can be evaluated at several points of therapy. ALT determinations are made at least every 3 months to confirm the biochemical response. ALT values are usually higher than those of AST. 

However, when the disease progresses to cirrhosis, the ratio can be reversed. In the table below, we see average values of ALT of 83.5 U/L in those who are unresponsive to treatment, which are much higher than those with treatment response (43.5 U/L). 

**Figure 1:**
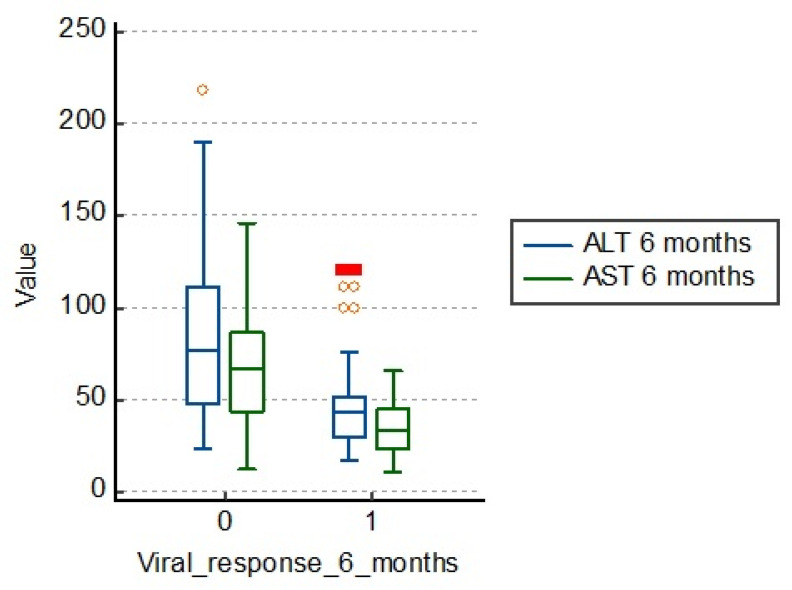
Distribution of the study group according to cytolysis enzyme values. ALT: alanine aminotransferase; AST: aspartate aminotransferase.

The same is observed about AST, the average being 66.0 U/L in those with therapeutic failure and 36.2 U/L in those responding to treatment. We can say that there is a significant correlation between cytolysis enzyme values and 6 months viral response in patients with chronic hepatitis B, as shown in Figure 1 and Table 2.

### 3.2. Influence of Viremia Values at 6 Months

The virological response is defined as a concentration of HBV-DNA less than 2log10 IU/mL. It is usually evaluated at 6 months, as well as 6 and 12 months after the end of treatment.

**Figure 2:**
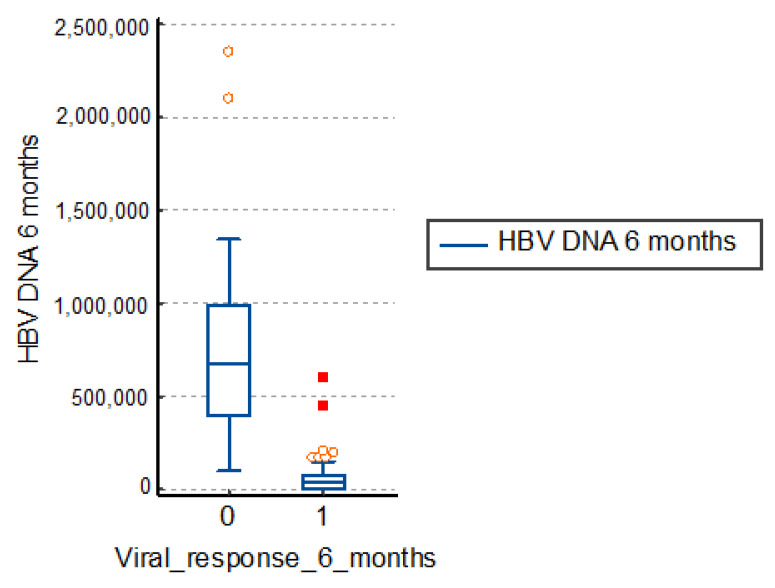
Distribution of viremia at 6 months. HBV: hepatitis B virus.

The study group patients with therapeutic failure had an average viremia after 6 months of treatment of 731,818.0 IU/mL, a much higher value than those with a positive response to treatment of 57,074.8 IU/mL. The differences are significant, so, in conclusion, a high viremia corresponds to a therapeutic failure, as in Table 3 and Figure 2.

### 3.3. Influence of BMI and Weight Values

**Figure 3:**
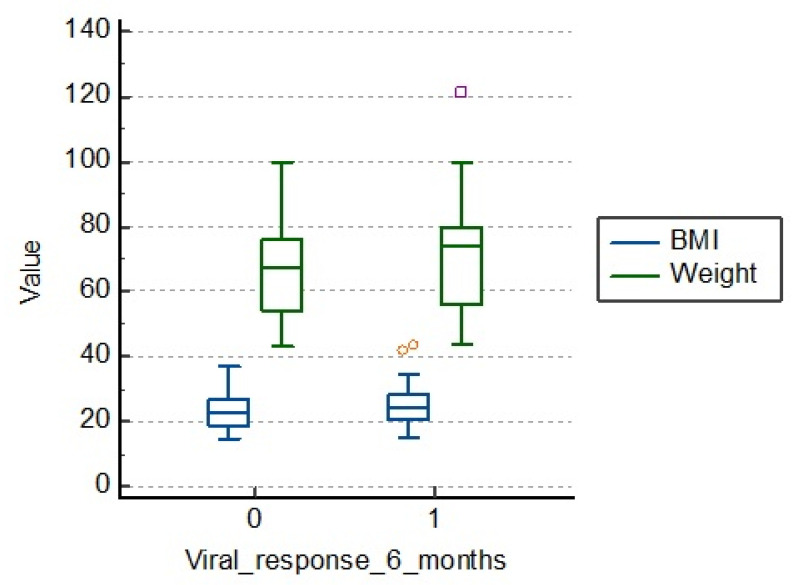
Distribution of BMI (body mass index) and weight at 6 months.

There is no significant influence of body mass index and weight compared to viral response at 6 months, as we also see from Table 4 and Figure 3. The average body mass index (BMI) in those with therapeutic failure is 23.2, and in others, 24.6. The average weight in those who had a viral response after 6 months is 70.6 kg, compared to those with therapeutic failure being 65.7 kg.

### 3.4. Influence of Factors Related to Carbohydrate Metabolism (Fasting Glucose, Fasting Insulin, HOMA-IR Index)

**Figure 4:**
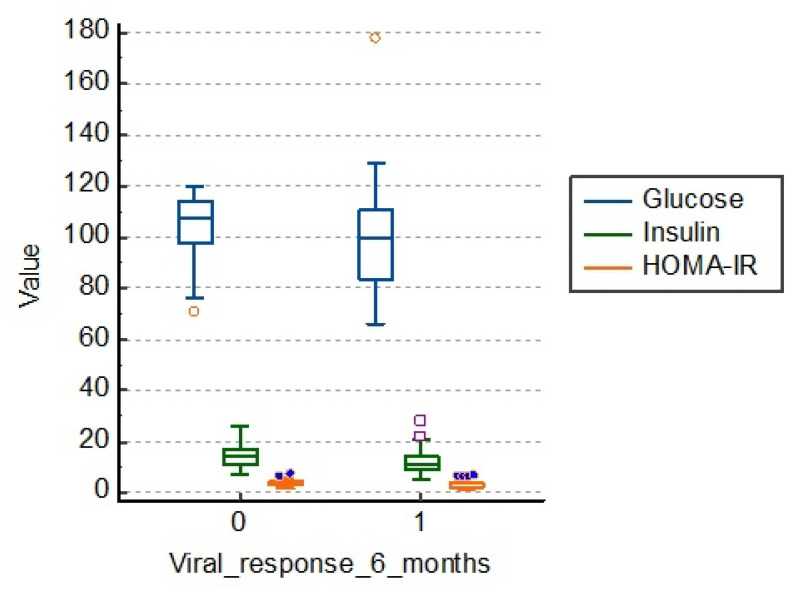
Distribution of the lot in relation to carbohydrate metabolism. HOMA-IR: homeostasis model of insulin resistance.

Patients in the study group who had a therapeutic failure had an average fasting blood glucose of 105.0 mg/dL, fasting insulinemia of 14.3 and the HOMA-IR index of 3.7. Those who had a viral response 6 months after treatment had lower average values, as shown in Table 5 and Figure 4.

There is a statistical significance regarding the link between carbohydrate metabolism and viral response to treatment at 6 months (*p* = 0.001), as shown in Figure 4.

### 3.5. Influence of the Presence of Metabolic Syndrome on 6 Months’ Viral Response 

From the table below, we can see that of the 129 subjects with absent metabolic syndrome only 31 of them had no response to treatment, whereas in those with present metabolic syndrome 18 of them had viral response at 6 months, as shown in Table 6.

**Figure 5:**
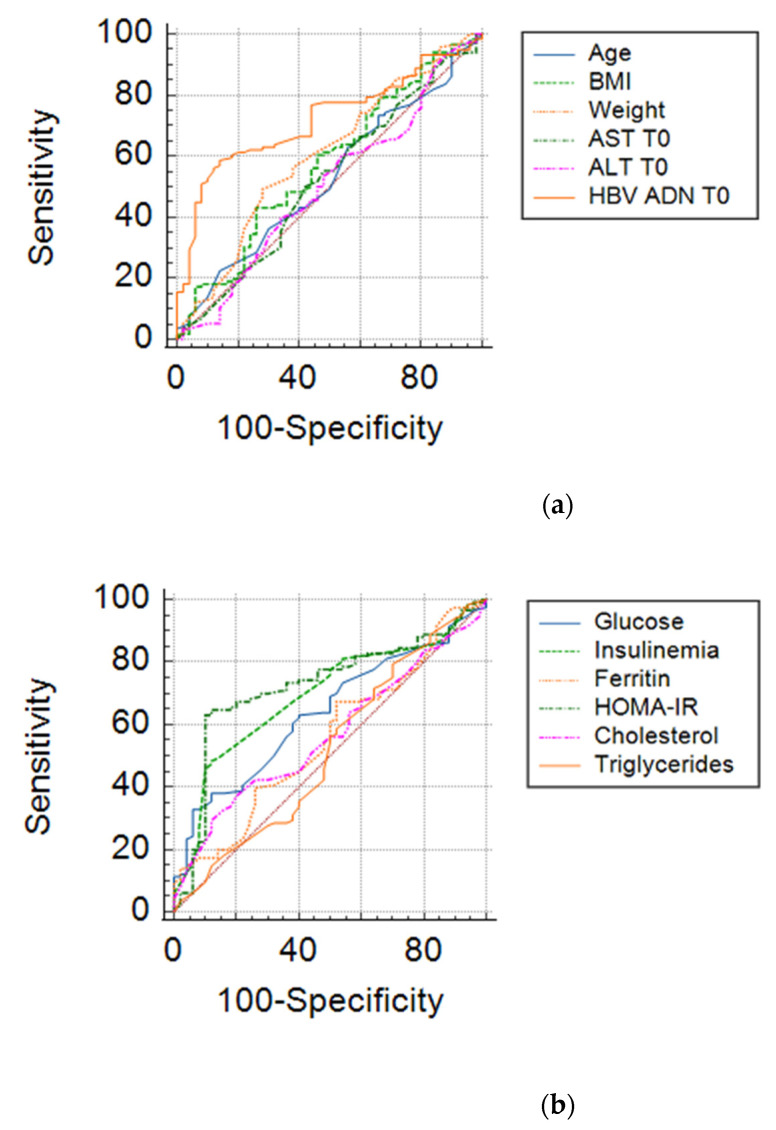
ROC curves for clinical-biochemical parameters: (**a**) Age, Weight, BMI, ALT T0, AST T0, HBV-DNA; (**b**) Glucose, Insulinemia, Ferritin, HOMA-IR, Cholesterol, Triglycerides.

We also performed a research related to the influence of clinical and biochemical parameters included in an analysis type AUC (area under curve) in order to estimate their degree of influence on the viral response after 6 months. The results are shown in Table 7 and Figure 5.

From the overall analysis of the above results, it appears that only HOMA index values, that of fasting insulin, together with baseline HBV DNA, ALT values, mean blood glucose at the beginning of treatment may be predictive of the early viral response in chronic hepatitis B. The limit values of the parameters that could anticipate the therapeutic success in chronic hepatitis B are shown in Table 8.

## 4. Discussion

Evaluation of potential parameters that may have a predictive value for the development of chronic hepatitis B showed that there are a number of factors that can interfere with the viral response. Viral load, liver iron score and insulin resistance index seem to have a significant degree of predictability in terms of viral response to therapy in chronic hepatitis B. Indeed, patients with high viral load and high degree of insulin resistance were less likely to achieve a virological response in chronic hepatitis B treated with Peg IFN, regardless of age, BMI, hepatic cytolysis enzymes, cholesterol and triglycerides.

Transabdominal ultrasound identifies steatosis only when it exceeds 20–30%, but it does not differentiate it from fibrosis. Fatty liver is a big liver, with increased echogenicity (big, white and bright liver), with posterior attenuation [14].

The performance of US B-mode imaging for the detection of mild steatosis (fat content > 5%) is low, with reported sensitivity of 60.9–65% [15,16]. A meta-analysis has assessed that, for the detection of moderate–severe fatty liver (>20–30% steatosis), B-mode US has a performance similar with computed tomography or magnetic resonance imaging (MRI). Compared to histology as reference standard, the overall sensitivity and specificity of B-mode US were, respectively, 84.8% and 93.6%, with 0.93 (0.91–0.95) area under the ROC curve (AUC-ROC) [17,18,19].

Levels of fasting blood glucose, fasting insulin, triglycerides, cholesterol, alanine-aminotransferase, aspartate-aminotransferase, HBV-DNA, body mass index, HOMA-IR and pathological changes in the liver with inflammation, steatosis were examined in all patients. Levels of BMI, HOMA-IR, fasting glucose, fasting insulin, triglycerides and cholesterol were significantly higher in patients with steatosis than those without steatosis, but levels of ALT, AST and HBV-DNA were significantly lower in patients with steatosis. Logistical regression analysis showed that only fasting insulin was a significant predictor for hepatic steatosis.

There is little knowledge about steatosis in chronic viral hepatitis B; various groups pointing out conflicting conclusions on these issues, ranging from a presumptive “protective effect” of steatosis as a result of treatment in patients with chronic hepatitis B, to “no effect” or a “harmful effect” as in the case of chronic hepatitis C.

There are studies that deny any interference between steatosis and viral response, considering that hepatic steatosis is only a common relative finding in chronic hepatitis B and that metabolic host factors, rather than viral factors, are responsible for the presence of hepatic steatosis in these patients. 

Minakari [20] investigated the prevalence of hepatic steatosis in a group of 132 patients with chronic HBV Infection Steatosis, which was present in 42.4% of patients and was not associated with age, sex, HBeAg, viral load, fibrosis score, serum cholesterol level, aspartate amino transferase, alanine amino transferase and alkaline phosphatase

Zheng [21] explored the clinical and virological characteristics of 360 patients with chronic hepatitis B with hepatic steatosis divided into two groups: a group with hepatosteatosis and a second group without hepatic steatosis. Body mass index, waist-to-hip ratio, fasting blood sugar, triglycerides, total cholesterol, transaminases, glutamyl-transpeptidase range, HBeag, viral load HBV-DNA and histological liver changes were compared between the two groups in order to assess the association of these factors with hepatic steatosis.

The group concluded that body mass index, waist-hip ratio, fasting glucose, triglycerides and cholesterol appear to be influence factors in chronic hepatitis B in combination with hepatic steatosis. However, hepatic steatosis in patients with chronic hepatitis B has been closely linked to changes in anthropometric indices and metabolic factors, but not to HBV infection.

Shi et al. [22] also considered that hepatic steatosis does not affect viral response, but affects biochemical response in patients with chronic hepatitis B treated with PEG-IFN.

The Lesmana Group [23] conducted a cross-study of patients with chronic hepatitis B to assess the prevalence of hepatic steatosis in chronic hepatitis B and to assess whether or not hepatic steatosis is associated with disease progression. Liver steatosis has been found in 30% of patients with chronic viral hepatitis B and is commonly associated with obesity.

A descriptive statistical analysis of the studied groups was developed for a better understanding of the clinical profile of patients and in order to initiate optimal therapy. The factors involved in therapeutic failure were: advanced age, increased viral load, insulin resistance.

## 5. Conclusions

The findings of our study were the following:

- The index HOMA, serum insulin levels alongside baseline HBV-DNA levels, baseline mean blood glucose, steatosis score and liver iron score may have a predictive value for obtaining an early viral response in chronic hepatitis B.
- The predictive factors of a favorable response in patients with chronic hepatitis B treated with α-IFN are represented by younger age, serum levels of ALT and HBV- DNA levels.
- Patients with high viral load and high degree of insulin resistance were less likely to acquire a virological response in chronic hepatitis B treated with Peg-IFN, regardless of age, BMI, hepatic cytolysis enzymes, cholesterol and triglycerides.
- There are a number of factors that can interfere with the viral response. Viral load, liver iron score and insulin resistance index seem to have a significant degree of predictability in regard of the viral response to the treatment in chronic hepatitis B. Thus, patients with high viral load and high degree of insulin resistance were less likely to acquire a virological response in chronic hepatitis B treated with Peg-IFN, regardless of age, BMI, hepatic cytolysis enzymes, cholesterol and triglycerides.

## Figures and Tables

**Table 1:** The synoptic characterization of the group. BMI: body mass index; ALT: alanine aminotransferase; AST: aspartate aminotransferase; HBV: hepatitis B virus.

	Women (*N* = 72)		Men (*N* = 94)		
	Mean	95% CI	Mean	95% CI	*p*
Age (year)	44.361	41.369–47.354	45.372	42.919–47.825	0.689
Fasting glucose (mg/dL)	101.653	98.108–105.197	98.074	94.427–101.722	0.09
Fasting insulin (µU/mL)	13.231	12.071–14.390	11.867	11.008–12.726	0.07
Weight (kg)	66.972	63.603–70.341	70.798	67.855–73.741	0.14
BMI (kg/m^2^)	23	22.502–25.161	24.515	23.96–25.635	0.5
ALT T0 (U/L)	154	136.654–172.512	143.138	129.039–157.238	0.26
AST T0 (U/L)	135	118.986–152.847	119.085	105.924–132.246	0.06
HBV DNA T0 (UI/mL)	3,425,458	2,692,369–4,158,547	3,681,361	2,984,874–4,377,848	0.80

**Table 2:** Evaluation of cytolysis enzymes.

	*N*	Median AST	Median ALT	*p*
Viral response months = 0	50	66	83.5	<0.001
Viral response months = 1	116	36.2	43.5	

**Table 3:** Viremia at 6 months.

	*N*	Median HBV-DNA 6 Months (UI/mL)	*p*
Viral response 6 months = 0	50	731,818	<0.001
Viral response 6 months = 1	116	57,074.8	

**Table 4:** Characterization of the lot in terms of BMI and weight.

	*N*	Median BMI (kg/m^2^)	Median Weight (kg)	*p*
Viral response 6 months = 0	50	23.2	65.7	0.7
Viral response 6 months = 1	116	24.6	70.6	

**Table 5:** Characterization of the lot in terms of carbohydrate metabolism. HOMA-IR: homeostasis model of insulin resistance.

	*N*	Median Fasting Glucose (mg/dL)	Median Fasting Insulin	Median HOMA_IR	*p*
Viral response 6 months = 0	50	105.0	14.3	3.7	0.001
Viral response 6 months = 1	116	97.3	11.6	2.8	

**Table 6:** Characterization of the patients’ lot in terms of metabolic syndrome.

	Metabolic Syndrome Absent	Metabolic Syndrome Present	Total
Viral response 6 months = 0	31 (24.03%)	19 (51.35%)	50 (30.1%)
Viral response 6 months = 1	98 (75.97%)	18 (48.65%)	116 (69.9%)
Total	129 (77.7%)	37 (22.3%)	166

**Table 7:** The interpretation of the lot through AUC analysis.

	AUC	St. Error	95% CI	Predictive Value
HOMA_IR	0.724	0.0428	0.640–0.808	YES
Initial value of HBV-DNA	0.721	0.0397	0.643–0.799	YES
Fasting insulin	0.694	0.0426	0.610–0.777	YES
Fasting glucose	0.638	0.0440	0.552–0.725	YES
Weight	0.603	0.0484	0.508–0.697	NO
Serum cholesterol	0.597	0.0474	0.504–0.690	NO
BMI	0.574	0.0488	0.478–0.670	NO
Serum ferritin	0.557	0.0480	0.463–0.651	NO
Age	0.528	0.0480	0.434–0.622	NO
AST	0.521	0.0498	0.423–0.619	NO
Serum Triglyceride	0.514	0.0499	0.416–0.611	NO
ALT	0.504	0.0498	0.406–0.601	NO

**Table 8:** Values of the parameters with predictive role for the therapeutic success.

Parameter	Optimal Value	95% CI	SE (%)	SP (%)	+LR (%)	−LR (%)	PPV (%)
HOMA_IR	≤2.64	2.37–2.91	64.66	88.00	5.39	0.40	69.70
Initial value of HBV-DNA	≤170,000	79,000–170,000	96.55	94.00	16.09	0.037	87.33
Level of serum insulin at the beginning of treatment	≤15.8	11–21	81.03	46.00	1.5	0.41	39.13
Level of serum glucose at the beginning of treatment	≤178	110–178	100.00	0.00	1	-	-

CI: Confidence interval; SE: Sensitivity; SP: Specificity; +LR: Likelihood positive ratio; −LR: Likelihood negative ratio; PPV: Positive predictive value.

## Data Availability

Not applicable.

## References

[1] Guvenir M., Arikan A. (2020). Hepatitis B Virus: From Diagnosis to Treatment. Pol. J. Microbiol..

[2] WHO Hepatitis B Key Facts. https://www.who.int/news-room/fact-sheets/detail/hepatitis-b.

[3] Jefferies M., Rauff B., Rashid H., Lam T., Rafiq S. (2018). Update on global epidemiology of viral hepatitis and preventive strategies. World J. Clin. Cases.

[4] Terrault N.A., Lok A.S., McMahon B.J., Chang K.-M., Hwang J., Jonas M.M., Brown R.S., Bzowej N.H., Wong J.B. (2018). Update on prevention, diagnosis, and treatment of chronic hepatitis B: AASLD 2018 hepatitis B guidance. Hepatology.

[5] Kim B.H., Kim W.R. (2018). Epidemiology of Hepatitis B Virus Infection in the United States. Clin. Liver Dis..

[6] CDC Interpretation of Hepatitis B Serological Tests Results. https://www.cdc.gov/hepatitis/hbv/index.htm.

[7] Zhang J., Lin S., Jiang D., Li M., Chen Y., Li J., Fan J. (2019). Chronic hepatitis B and non-alcoholic fatty liver disease: Conspirators or competitors?. Liver Int..

[8] Colangelo L.A., Gapstur S.M., Gann P.H., Dyer A.R., Liu K. (2002). Colorectal cancer mortality and factors related to the insulin resistance syndrome. Cancer Epidemiol. Biomark. Prev..

[9] Chen C., Yang H., Yang W.-S., Liu C.-J., Chen P.-J., You S., Wang L., Sun C., Lu S., Chen D.-S. (2008). Metabolic Factors and Risk of Hepatocellular Carcinoma by Chronic Hepatitis B/C Infection: A Follow-up Study in Taiwan. Gastroenterology.

[10] Forţofoiu M., Forţofoiu M.C., Comănescu V., Dobrinescu A.C., Pădureanu V., Vere C.C., Streba C.T., Ciurea P.L. (2015). Hepatocellular car-cinoma and metabolic diseases—histological perspectives from a series of 14 cases. Rom. J. Morphol. Embryol..

[11] Senoymak M.C., Ozkan H. (2020). Evaluation of the Relationship between Insulin Resistance and HBV DNA Level in Patients with HBeAg-negative Chronic HBV Infection (Natural Course Phase 3). Euroas. J. Hepatogastroenterol..

[12] Niederau C., Heintges T., Lange S., Goldmann G., Niederau C.M., Mohr L., Häussinger D. (1996). Long-Term Follow-up of HBeAg-Positive Patients Treated with Interferon Alfa for Chronic Hepatitis, B. N. Engl. J. Med..

[13] Lok A.S.F., McMahon B.J. (2009). Chronic hepatitis B: Update 2009. Hepatology.

[14] Popescu M., Popescu I.A., Stanciu M., Cazacu S.M., Ianoşi N.G., Comănescu M.V., Singer C.E., Neagoe C.D. (2016). Non-alcoholic fatty liver disease—clinical and histopathological aspects. Rom. J. Morphol. Embryol..

[15] Kim S.H., Lee J.M., Kim J.H., Kim K.G., Han J.K., Lee K.H., Park S.H., Yi N.-J., Suh K.-S., An S.K. (2005). Appropriateness of a Donor Liver with Respect to Macrosteatosis: Application of Artificial Neural Networks to US Images—Initial Experience. Radiology.

[16] Hernaez R., Lazo M., Bonekamp S., Kamel I., Brancati F.L., Guallar E., Clark J.M. (2011). Diagnostic accuracy and reliability of ultrasonography for the detection of fatty liver: A meta-analysis. Hepatology.

[17] Dasarathy S., Dasarathy J., Khiyami A., Joseph R., Lopez R., McCullough A.J. (2009). Validity of real time ultrasound in the diagnosis of hepatic steatosis: A prospective study. J. Hepatol..

[18] Van Werven J.R., Marsman H.A., Nederveen A.J., Smits N.J., Kate F.J.T., Van Gulik T.M., Stoker J. (2010). Assessment of Hepatic Steatosis in Patients Undergoing Liver Resection: Comparison of US, CT, T1-weighted Dual-Echo MR Imaging, and Point-resolved1H MR Spectroscopy. Radiology.

[19] Ferraioli G., Monteiro L.B.S. (2019). Ultrasound-based techniques for the diagnosis of liver steatosis. World J. Gastroenterol..

[20] Minakari M., Molaei M., Shalmani H.M., Alizadeh A.H.M., Jazi A.H.D., Naderi N., Shavakhi A., Mashayekhi R., Zali M. (2009). Liver steatosis in patients with chronic hepatitis B infection: Host and viral risk factors. Eur. J. Gastroenterol. Hepatol..

[21] Zheng R.-D., Chen J.-N., Zhuang Q.-Y., Lu Y.-H., Chen J., Chen B.-F. (2013). Clinical and Virological Characteristics of Chronic Hepatitis B Patients with Hepatic Steatosis. Int. J. Med. Sci..

[22] Shi J.P., Lu L., Qian J.C., Ang J., Xun Y.H., Guo J.C., Shi W.L., Wang Y.F., Fan J.G. (2012). Impact of liver steatosis on antiviral effects of pegylated interferon-alpha in patients with chronic hepatitis B. Chin. J. Hepatol..

[23] Lesmana L.A., Lesmana C.R., Pakasi L.S., Krisnuhoni E. (2012). Prevalence of hepatic steatosis in chronic hepatitis B patients and its association with disease severity. Acta Med. Indon..

